# Postoperative Ileus with the Topical Application of Tongfu Decoction Based on Network Pharmacology and Experimental Validation

**DOI:** 10.1155/2022/2347419

**Published:** 2022-03-28

**Authors:** Ximeng Zuo, Xiaoguang Shi, Xuedan Zhang, Zhenzhou Chen, Zhenrui Yang, Xiaojuan Pan, Rui Lai, Ze Zhao

**Affiliations:** ^1^Department of General Surgery, Beijing University of Chinese Medicine Dongzhimen, No. 5 Shipping Warehouse, Dongcheng District, Beijing 100700, China; ^2^Department of Chinese Medicine, Beijing Daxing District People's Hospital, 26 Huangcun West Street, Daxing District, Beijing, China

## Abstract

**Objective:**

Postoperative gastrointestinal dysfunction is a common and important complication of surgery. This study aimed to explore the key pharmacological mechanisms of Tongfu decoction in treating postoperative ileus (POI).

**Methods:**

The active ingredients of Tongfu decoction and their targets were screened using the TCMSP database and STITCH and SwissTargetPrediction databases, respectively. The GeneCards and DisGeNET databases were used to obtain POI dysfunction-related therapeutic targets. After screening, a drug-active-ingredient-therapeutic target network was constructed and the key target functional enrichment analysis was carried out. The Sprague–Dawley rats with POI were used for *in vivo* experimental verification. The serum levels of IL-1*β*, IL-6, IL-10, IFN-*γ*, and MCP-1 were measured after surgery using enzyme-linked immunosorbent assay. The Western blot analysis was used to determine the expression of key proteins of the PI3K-Akt signaling pathway in colon tissues.

**Results:**

An interaction network was constructed containing 7 Chinese medicine components, 36 compounds, and 85 target proteins. The functional enrichment analysis showed that the target proteins mainly acted on the POI through the PI3K-Akt signaling pathway. In *in vivo* experiments, Tongfu decoction had a promoting effect on the serum level of IL-10, an inhibitory effect on the serum levels of IL-1*β* and CCL2, and an inhibitory effect on the local expression of PI3K, pAkt, and mTOR in colon tissue. In addition, the Tongfu decoction increased the intestinal ink advancing rate.

**Conclusion:**

Nonoral Tongfu decoction can also be used to treat POI; its mechanism is affected by IL-10 and IL-1*β*.The inhibition of the PI3K-Akt signaling pathway affected the treatment with Tongfu decoction by inducing an immune-inflammatory storm in POI.

## 1. Introduction

In this study, the key pharmacological mechanism of Tong fu decoction, a traditional Chinese medicine (TCM), in treating postoperative gastrointestinal dysfunction was mainly explored through network pharmacology and experimental verification. Postoperative ileus (POI) is one of the major postoperative complications of gastrointestinal surgery. Its etiology has been mainly classified into two stages. The early stage is 3–6 h after surgery, during which sympathetic inhibition is induced by surgical stimulation. The second stage is 6–72 h after surgery, during which an immune mediator-driven inflammation occurs [1]. If the patient is unable to recover within 120 h, these complications may lead to more serious consequences [2] and even death.

At present, the main way to prevent and treat POI is the promotion of enhanced recovery surgery (ERAS). ERAS was proposed by Kehlet in the 1990s, and it has been continuously optimized and improved. ERAS is a multidisciplinary, individualized diagnosis and treatment discipline involving a team of surgeons, anesthesiologists, and nursing staff. Its main purpose is to speed up the postoperative rehabilitation process and reduce the incidence of postoperative complications [3]. The content of ERAS in modern medicine includes fasting for 2 h before surgery, application of minimally invasive surgery, improvement in anesthesia plan, liquid management of postoperative parenteral nutrition, reasonable drainage tube removal, and preoperative and postoperative missions [4]. The main purpose of this treatment is to shorten the length of hospitalization and reduce the cost of hospitalization and incidence of postoperative complications, but the problem of poor compliance still exists.

In previous studies, TCM promoted the recovery of patients [5–11] and had a synergistic effect on ERAS [12–15]. One of the drugs used was Tongfu decoction, having a certain effect on POI using nonoral treatment methods such as rectal instillation, which could accelerate the recovery of postoperative gastrointestinal function and relieve postoperative inflammation of the digestive tract [16–19]. The theory of TCM believes that the treatment of Tongli Gongxia, which lowers the adversity and regulates qi, can relieve abdominal distension, nausea, and vomiting in patients with POI. Also, it promotes postoperative defecation and inhibits inflammation [20]. Despite some promising clinical evidence, the pharmacological mechanism of Tongfu decoction in treating POI remains unexplored.

## 2. Methods

### 2.1. Network Pharmacology

#### 2.1.1. Screening of Active Compounds in Compound Prescriptions

The TCMSP database, which is a pharmacological database and analysis platform of the Chinese medicine system (http://lsp.nwu.edu.cn/tcmsp.php), was used in this study. The database included 499 Chinese medicines registered in the Chinese Pharmacopoeia, containing 29,384 ingredients, 3311 targets, and 837 related diseases. The disease information in this database comes from the TTD and PharmGKB databases. The active ingredients of Rhubarb, *Magnolia officinalis*, Fructus Aurantii, *Angelica sinensis*, Toosendan Fructus, and Aucklandiae Radix were searched in the TCMSP database using OB and DL as indicators, ADME as a comprehensive model, and oral bioavailability of OB ≥ 30% and drug similarity DL ≥ 0.18 as screening thresholds.

#### 2.1.2. Screening of Active Compound Targets

STITCH (https://stitch.embl.de/) and SwissTargetPrediction databases were used to screen the targets of active compounds of Tongfu decoction obtained in Section 2.1.1. The PubChem database (https://pubchem.ncbi.nlm.nih.gov/) was used to add all the active compounds to the STITCH database. The compound was converted into the standard canonical SMILES format and imported into SwissTargetPrediction. The target with parameter probability >0 was selected for further analysis.

#### 2.1.3. Construction of Pharmacologically Active Compound-Target Protein Network

Based on the results of the target proteins of the pharmacologically active compounds screened using STITCH and SwissTargetPrediction, Cytoscape was used to construct the “pharmacologically active compound-target protein network” in the compound.

#### 2.1.4. Screening for Targets of POI

GeneCards (https://www.drugBank.ca/) and DisGeNET (https://www.disgenet.org/) were used to uncover the genes that affected POI. The intersection targets of genes that affected POI and the target protein of the compound with drug activity were the key research objects.

#### 2.1.5. Constructing Key Target PPI Networks

A key target PPI network was constructed using the STRING (https://stringdb.org) website to understand the functions and connections of the aforementioned proteins. Cytoscape completed the drawing and beautification of the network.

#### 2.1.6. GO and KEGG Enrichment Analyses

GO enrichment and KEGG analyses on the intersection genes were conducted in the R software clusterProfiler package. Pathview was used to visualize the KEGG pathway. GO enrichment analysis mainly described the biological processes (BP), cellular components (CC), and molecular functions (MF) correlated with genes.

### 2.2. Experiment

#### 2.2.1. Composition of Tongfu Decoction

The external preparation of Tongfu decoction comprised Chinese medicine decoction pieces. These pieces were selected by Beijing Tongrentang Pharmaceuticals. The specific ingredients were as follows: Rhubarb, *M. officinalis*, Fructus Aurantii, *A. sinensis*, radish seeds, Toosendan Fructus, and Aucklandiae Radix. The aforementioned drug was crushed and mixed with petroleum jelly. The weight ratio of drug:petroleum jelly was 1 : 2 to make a plaster with a weight of 5 g.

#### 2.2.2. Animal Experiments

The experimental animals were healthy male Sprague–Dawley rats, aged 9–10 weeks, and weighing 200 ± 10 g, which were routinely reared. These rats were divided into model (small intestine surgery) group, negative control (abdominal incisions only) group, Tongfu decoction group, neostigmine group, and blank group. Each group was randomly divided into postoperative 24-h, 72-h, and 120-h groups. The rats in each group were fasted for 24 h before surgery and allowed to drink water ad libitum. The method of modeling was an aseptic operation, which involved opening the abdominal cavity via a midline incision of the abdomen, turning the small intestine to the left, placing it on the gauze soaked with normal saline, and squeezing from the dactyly to the ileocecal area. This step was to simulate the clinically common whole small-bowel explorative surgery. The rats in the negative control group only underwent opening of the abdomen and intestines without pushing operation. Then, the small intestine was returned in order, a small amount of saline was sprinkled into the abdominal cavity, and the abdominal cavity was closed with intermittent sutures.

After the surgery, the rats were subcutaneously injected with 20,000 IU/kg penicillin G sodium and 250 mg/kg streptomycin sulfate on the back for three consecutive days. In the neostigmine group, 10 *µ*g of neostigmine mesylate was subcutaneously injected daily. The Tongfu decoction was applied to the scapular skin of the rats in the form of a patch and fixed with tape. The dressing was changed every 24 h. After the surgery, the rats were fasted for 48 h without water and given 20% glucose solution twice in the morning and evening at an interval of 8 h. On the third day after the surgery, 20 mL/kg feed paste was fed (standard feed was superfinely pulverized, and 20% feed paste was prepared). The diet was provided free on the fourth day.

#### 2.2.3. Enzyme-Linked Immunosorbent Assay, Western Blot, and Hematoxylin and Eosin Staining

The serum levels of IL-1*β*, IL-6, IL-10, IFN-*γ*, and MCP-1 (Proteintech, China) in rats were detected by enzyme-linked immunosorbent assay (ELISA) after 1 day, 3 days, and 5 days of the surgery. A BCA protein determination kit (BioSharp, China) was used to determine the total protein content of rat colon tissue 24 h after the surgery. An equal amount of protein was separated using a 10% SDS-PAGE gel and transferred to a polyvinylidene fluoride membrane (Millipore, USA). After blocking, the membrane was incubated with the corresponding primary antibodies (PI3K, Akt, pAkt, and mTOR) at 4°C overnight. After washing with TBST, the membrane was incubated with the secondary antibody for 1.5 h. The villus ratio of all antibodies was 1 : 5000 (Proteintech, USA). Western blot detection used ECL chemiluminescence detection (BioSharp, China) and Amersham imager600 (Tianneng, China). The colon tissue of the rat was taken 24 h after the surgery, fixed with 10% formalin, embedded and cut into 4-*μ*m sections, and stained with hematoxylin and eosin (HE); the tissue morphology was observed.

#### 2.2.4. Statistical Analysis

The data between groups were analyzed using one-way analysis of variance (SPSS 28.0). The Student *t*-test was used for the data between the two groups. These results were presented as the mean ± standard error of the mean for the treated experimental and control groups. The *p* values were indicated as ^*∗*^*p* < 0.05, ^*∗∗*^*p* < 0.01, and ^*∗∗∗*^*p* < 0.001.

## 3. Results

### 3.1. Active Ingredients and Targets in Tongfu Decoction

The active ingredients corresponding to Rhubarb, *M. officinalis*, Fructus Aurantii, *A. sinensis*, Toosendan Fructus, and Aucklandiae Radix were uncovered by searching in the TCMSP database (Table 1)

### 3.2. Potential Target of Tongfu Decoction in Treating POI

**Figure 1:**
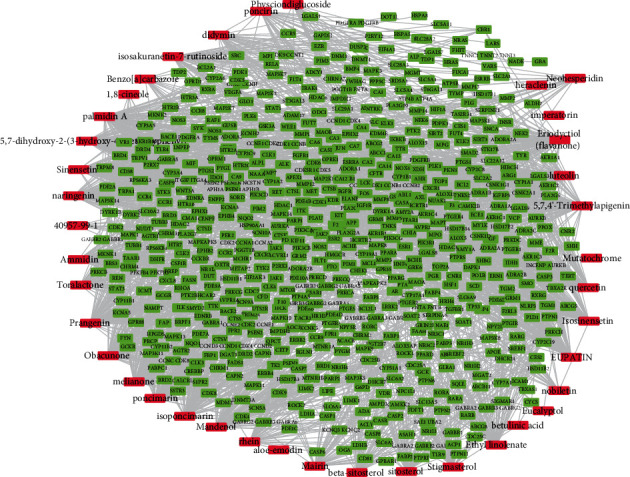
Interaction network between active pharmaceutical ingredients and target protein. (a) Green represents Tongfu decoction targets, while red represents active ingredients.

Based on the results of the target proteins of the pharmacologically active compounds screened by STITCH and SwissTargetPrediction, Cytoscape was used to construct a compound with pharmacological activity-target protein network, which contained 39 compounds and corresponding 649 target proteins (Figure 1(a)).

**Figure 2:**
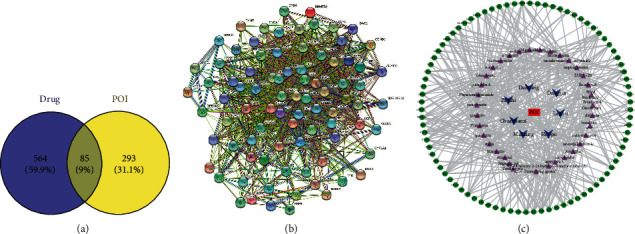
(a) Venn diagram showing the overlap of the targets of Tongfu decoction and POI. Blue represents Tongfu decoction targets, while yellow represents POI targets. (b) Target protein interaction network. The nodes in the network represent proteins, the lines show the proteins' functional associations, and the thickness of lines corresponds to the observed association's confidence level. (c) Drug-active-ingredient-target protein network. The blue triangle represents the drug, the purple triangle represents the active ingredient, and the green circle represents the target protein.

Then, the genes that affected POI were screened using GeneCards and DisGeNET, and the intersection with the target protein of the drug-active compound in Figure 1 was taken, obtaining 85 overlapping genes (Figure 2(a))

### 3.3. PPI Network Establishment

A PPI network based on 85 intersection genes was created using the STRING website (Figure 2(b)). Cytoscape was used to complete network drawing and beautification (Figure 2(c)). A drug-active ingredient-target protein network was further established (Figure 2(c)).

### 3.4. GO and KEGG Enrichment Analysis

**Figure 3:**
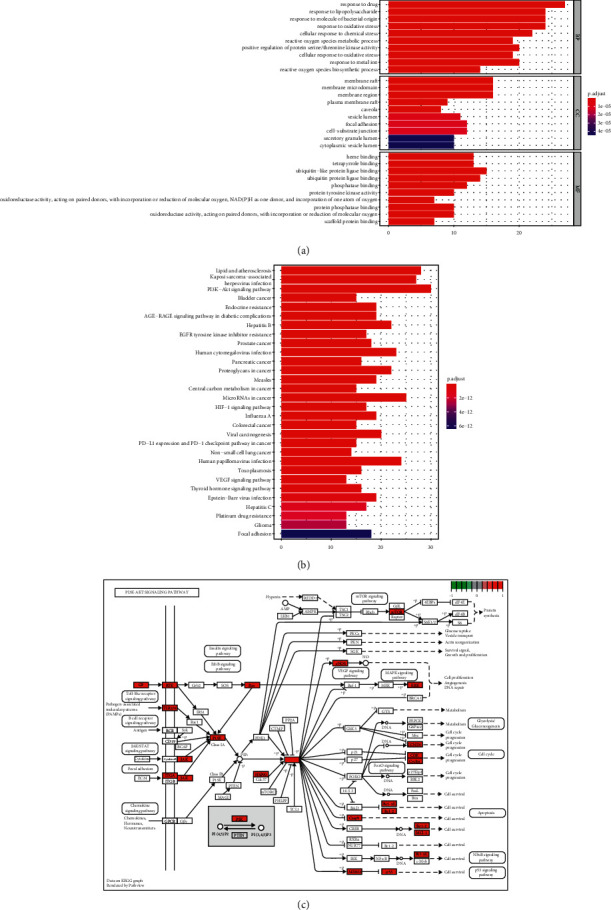
(a) GO enrichment analysis of target proteins. (b) KEGG enrichment analysis of target proteins. (c) Schematic of the PI3K-Akt signaling pathway.

GO enrichment analysis was performed on 85 intersection genes, and the most significantly enriched 10 GO terms in BP, CC, and MF categories were revealed, as shown in Figure 3(a). The top 5 GO BP terms were “response to drug,” “response to lipopolysaccharide,” “response to molecule of bacterial origin,” “response to oxidative stress,” and “cellular response to chemical stress.” KEGG enrichment analysis was also performed on the intersection genes, and the 30 most significant signaling pathways were identified, as listed in Figure 3(b). The parts closely related to inflammation were screened. Among these, the PI3K-Akt signaling pathway was significant for POI (Figures 3(b) and 3(c)).

### 3.5. Effect of Tongfu Decoction on the Intestinal Function of Rats with POI

**Figure 4:**
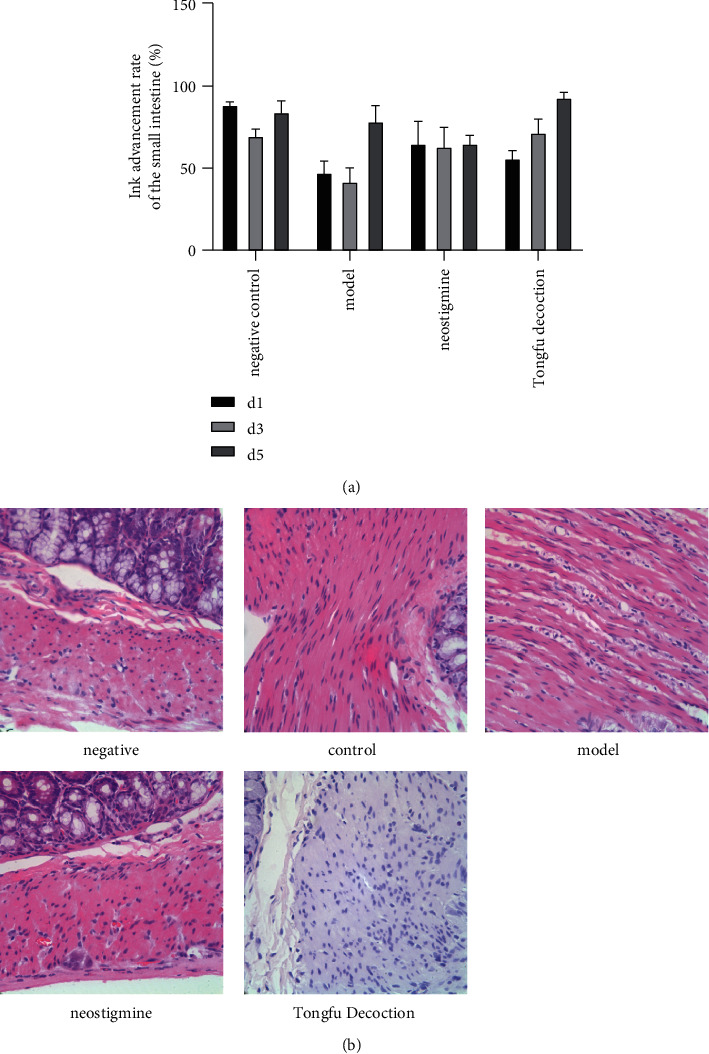
(a) Ink advancement rate of the small intestine. (b) HE staining of the colon tissue in different groups. Compared with the model group, rats treated with Tongfu decoction had more complete colonic mucosal epithelial structure and less infiltration of eosinophils, neutrophils, and lymphocytes in colon villi and muscle tissues and were less prone to vasodilation.

The pass rate of the intestine for rats with POI was measured to verify the therapeutic effect of Tongfu decoction on POI. The intestine passage rate was significantly higher in rats using Tongfu decoction than in those in the model and neostigmine groups on the third day after the surgery (*p* < 0.01), suggesting that Tongfu decoction had a certain effect on POI treatment (Figures 4(a) and 4(b)).

The rat colon tissue was HE stained. Rats using Tongfu decoction had a much more complete colonic mucosal epithelial structure and less infiltration of eosinophils, neutrophils, and lymphocytes in colon villi and muscle tissues. Also, they were less prone to vasodilation (Figure 4). These results showed that Tongfu decoction improved the inflammatory pathological changes that occurred after the surgery of the colon tissue in rats.

**Figure 5:**
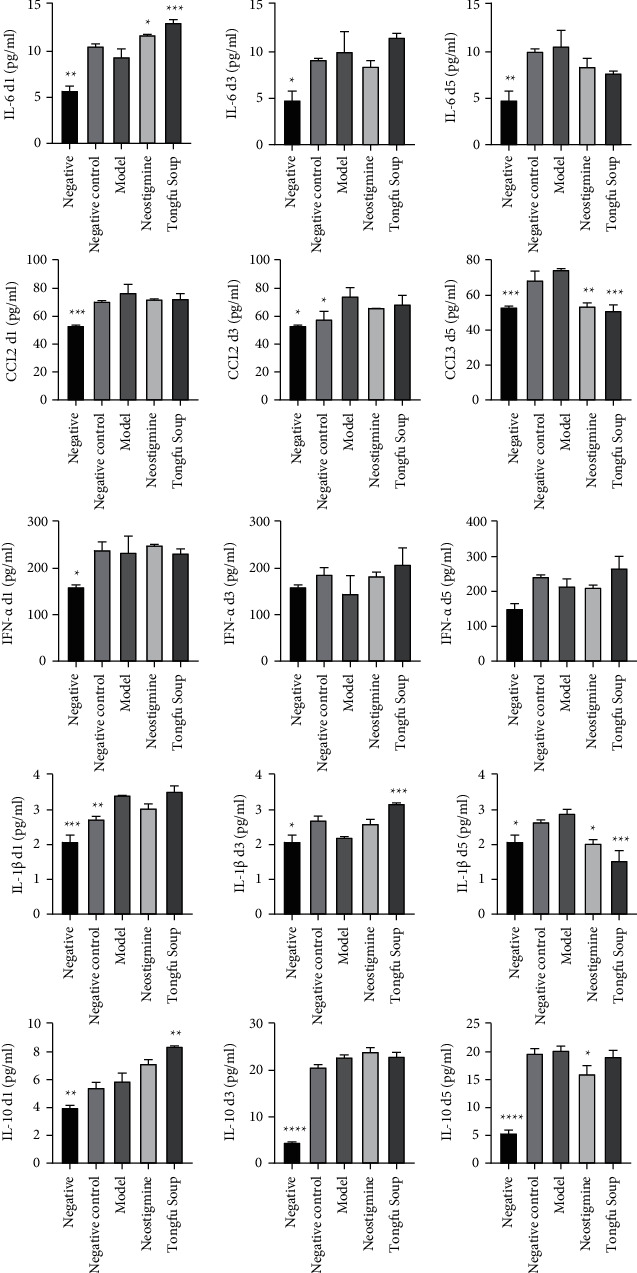
Effects of Tongfu decoction on the expression of IL-6, CCL2, and IFN in villi and muscle tissues and IL-10 (days 1, 3, and 5) after surgery (compared with the model group, ^*∗∗∗∗*^*p* < 0.0001, ^*∗∗∗*^*p* < 0.001, ^*∗∗*^*p* < 0.01, and ^*∗*^*p* < 0.05).

The rats using Tongfu decoction exhibited a significant increase in the serum level of IL-10 on the first day after the surgery (*p* < 0.01) and a significant decrease in the serum levels of CCL2 and IL-1*β* on the fifth day (*p* < 0.01) (Figure 5).

**Figure 6:**
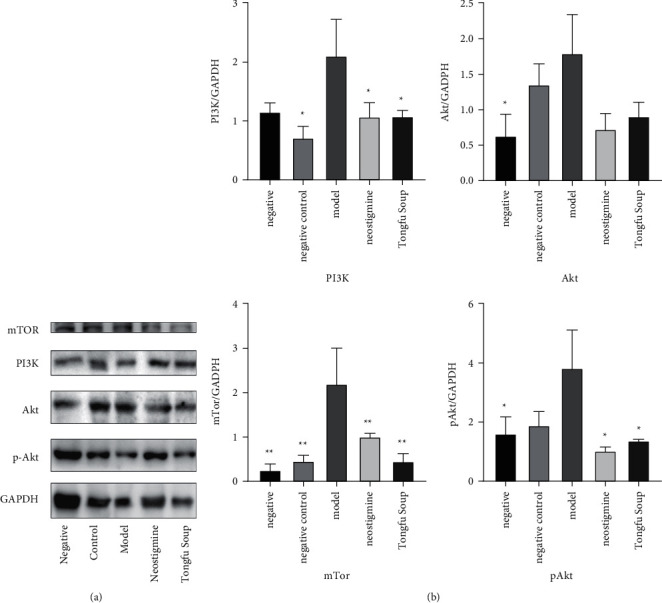
(a) Western blot showing the effects of Tongfu decoction on the expression of proteins related to the PI3K-Akt pathway in colon tissue. (b) Relative quantitative results of the Western blot (compared with the model group, ^*∗∗*^*p* < 0.01 and ^*∗*^*P* < 0.05).

The expression levels of PI3K, pAkt, and mTOR in the colon tissue of rats treated with Tongfu decoction reduced compared with those in the model group (*p* < 0.05) (Figure 6).

## 4. Discussion

The main components of Tongfu decoction are tannins, anthracene derivatives, stilbene derivatives, phenbutanone, and naphthalene derivatives. These components increase intestinal mucosal motility, inhibit intestinal water absorption, exert anti-inflammatory effects, and reduce cytokine secretion [21–23]. Glauber's salt is a natural mineral with Na_2_SO_4_·10H_2_O as the main component, which has anti-inflammatory and cathartic effects. The pharmacological effects of other drugs such as *M. officinalis* and *Citrus aurantium* are related to the improvement in intestinal motility.

This study was conducted to investigate the molecular mechanism of Tongfu decoction in treating POI. Based on the predicted targets of network pharmacology, this study focused on the effects of Tongfu decoction on different immune mediators and the PI3K-Akt signaling pathway. Rats with POI were used as experimental materials to explore the antagonistic effect of Tongfu decoction on the inflammatory response of postoperative colon tissue in rats and to verify the prediction results of network pharmacology. In this study, the network pharmacology methods were used to systematically analyze the TCM compounds in Tongfu decoction at the molecular level. A total of 39 potentially active Chinese medicine compounds, 649 target proteins related to postoperative gastrointestinal dysfunction, 85 potential genes, and 30 most relevant signaling pathways were obtained. According to the KEGG analysis, the signaling pathway with the highest correlation was the PI3K-Akt signaling pathway, suggesting that Tongfu decoction might play a role in treating POI by regulating the PI3K-Akt signaling pathway. This pathway was of great significance in the inflammatory response. This conclusion was confirmed in various experiments. *In vivo* experiments showed that external application of the Tongfu decoction patch after the surgery could increase the passage rate of small intestinal contents in rats. This meant that not only oral administration but also a topical patch of Tongfu decoction could promote the recovery of small intestinal function. Western blot analysis and ELISA revealed that Tongfu decoction treated the disease by affecting the mechanism of the PI3K-Akt signaling pathway. Tongfu decoction also reduced the serum levels of CCL2 and IL-1*β*, increased the serum level of IL-10 and reduced the infiltration of local eosinophils and monocytes in colon tissue.

Previous studies demonstrated a reduction in the levels of CCL2 and IL-1*β* and an increase in the level of IL-10 had positive effects on the treatment of POI. CCL2 is one of the key chemokines regulating monocyte/macrophage migration and infiltration. Its reduction can improve inflammation and POI [24]. As an inflammatory mediator, IL-1*β* directly affects the symptoms of POI (gas, defecation, nausea, vomiting, and so on). Its release depends on the number of AIM2 inflammasomes and microorganisms [25]. IL-10 is a potent anti-inflammatory mediator that reduces neutrophil infiltration, cytotoxicity [26], and inflammatory responses induced by intraoperative hemorrhage [27], regardless of sepsis-induced gastrointestinal function [28] or postoperative gastrointestinal dysfunction [29]. The serum levels of IL-10 peaked about 4 h after laparotomy and returned to normal levels within 3 days after the surgery [30]. In this study, the expression of IL-10 increased on the first day after the surgery, while the levels of CCL2 and IL-1*β* decreased on the third and fifth days after the surgery, suggesting that Tongfu decoction affected the early postoperative inflammatory response. The secretion of anti-inflammatory mediators had an inhibitory effect on the secretion of inflammatory mediators in the long-term intestinal tract.

The PI3K-Akt signaling pathway plays an important role in the inflammatory response. PI3K signaling promotes the inflammatory activity of T cells by phosphorylating the FOXO transcription factor [31]. In POI, an excessive inflammatory response is not required, and Tongfu decoction can reduce the activity of the PI3K-Akt signaling pathway. In BV2, LPS treatment did not affect the relative levels of PI3K and NF-*κ*B p65 expression but significantly increased the relative levels of phosphorylated Akt and NF-*κ*B p65, implying that pAkt overexpression might affect not only downstream inflammatory expression but also the nervous system, thereby inducing postoperative gastrointestinal dysfunction [33]. The factors such as CCL2 and IL-1*β* were in the PI3K-Akt-related downstream signaling pathway, which was consistent with the hypothesis that the regulation of PI3K-Akt signaling pathway could affect the development of inflammation in postoperative gastrointestinal dysfunction. Therefore, it was presumed that the main target of Tongfu decoction was the PI3K-Akt signaling pathway.

The comprehensive and systematic analysis of drugs-active-ingredients-target proteins in network pharmacology conforms to the holistic concept, and its research concept coincides with the overall concept of TCM syndrome differentiation and treatment. Network pharmacology is often used in the study of TCM components and targets [32–37], especially TCM compound prescriptions. Different chemical components in TCM compounds may have synergistic effects in terms of reducing toxicity. Therefore, the combination of TCM and network pharmacology bridges the gap between modern medicine and traditional medicine [38]. This study explored the mechanism of Tongfu decoction in treating postoperative gastrointestinal dysfunction and identified a new form of percutaneous topical administration. In previous studies, rectal instillation and jejunal injection were usually chosen for administration [39], which were associated with problems such as poor compliance and further irritation of the digestive tract.

The form of administration described in the present study provided greater compliance and was simpler than rectal instillation [39, 40]. Moreover, transdermal administration did not consider liver toxicity; only local allergic reactions were considered, and it was highly safe. In addition, these results suggested that the therapeutic effect of Tongfu decoction on gastrointestinal dysfunction after gastrointestinal surgery was probably exerted via inhibiting the inflammatory storm induced by the PI3K-Akt signaling pathway. The therapeutic mechanism might be the same. The pharmacological effects of the compound may also be applied to other acute abdominal diseases such as severe pancreatitis and acute peritonitis. Also, the indications can be further explored in subsequent studies.

## Figures and Tables

**Table 1:** Including 16 active ingredients for Rhubarb, 2 active ingredients for *M. officinalis*, 22 active ingredients for Fructus Aurantii, 2 active ingredients for *A. sinensis*, 3 ingredients for radish seeds, 9 active ingredients for Toosendan Fructus, and 6 active ingredients for Aucklandiae Radix, after deduplicating these 60 compounds, 56 different compounds were obtained eventually.

Mol ID	Molecule name	OB (%)	Caco-2	DL	Herb
MOL002235	EUPATIN	50.8	0.53	0.41	Dahuang
MOL002251	Mutatochrome	48.64	1.97	0.61	Dahuang
MOL002259	Physciondiglucoside	41.65	−2.64	0.63	Dahuang
MOL002260	Procyanidin B-5,3′-O-gallate	31.99	−1.61	0.32	Dahuang
MOL002268	Rhein	47.07	−0.2	0.28	Dahuang
MOL002276	Sennoside E_qt	50.69	−0.74	0.61	Dahuang
MOL002280	Torachrysone-8-O-beta-D-(6′-oxayl)-glucoside	43.02	−1.23	0.74	Dahuang
MOL002281	Toralactone	46.46	0.86	0.24	Dahuang
MOL002288	Emodin-1-O-beta-D-glucopyranoside	44.81	−1.12	0.8	Dahuang
MOL002293	Sennoside D_qt	61.06	−0.7	0.61	Dahuang
MOL002297	Daucosterol_qt	35.89	1.35	0.7	Dahuang
MOL002303	Palmidin A	32.45	−0.36	0.65	Dahuang
MOL000358	Beta-sitosterol	36.91	1.32	0.75	Dahuang
MOL000471	Aloe-emodin	83.38	−0.12	0.24	Dahuang
MOL000554	Gallic acid-3-O-(6′-O-galloyl)-glucoside	30.25	−1.96	0.67	Dahuang
MOL000096	(-)-Catechin	49.68	−0.03	0.24	Dahuang
MOL005970	Eucalyptol	60.62	1.1	0.32	Houpu
MOL005980	Neohesperidin	57.44	0.29	0.27	Houpu
MOL013276	Poncirin	36.55	−1.67	0.74	Zhishi
MOL013277	Isosinensetin	51.15	1.16	0.44	Zhishi
MOL013279	5,7,4′-Trimethylapigenin	39.83	1.01	0.3	Zhishi
MOL013428	Isosakuranetin-7-rutinoside	41.24	−1.59	0.72	Zhishi
MOL013430	Prangenin	43.6	0.8	0.29	Zhishi
MOL013433	Prangenin hydrate	72.63	0.14	0.29	Zhishi
MOL013435	Poncimarin	63.62	0.66	0.35	Zhishi
MOL013436	Isoponcimarin	63.28	0.5	0.31	Zhishi
MOL013437	6-Methoxy aurapten	31.24	1.01	0.3	Zhishi
MOL013440	Citrusin B	40.8	−1.94	0.71	Zhishi
MOL001798	Neohesperidin_qt	71.17	0.26	0.27	Zhishi
MOL001803	Sinensetin	50.56	1.12	0.45	Zhishi
MOL001941	Ammidin	34.55	1.13	0.22	Zhishi
MOL013352	Obacunone	43.29	0.01	0.77	Zhishi
MOL002914	Eriodyctiol (flavanone)	41.35	0.05	0.24	Zhishi
MOL004328	Naringenin	59.29	0.28	0.21	Zhishi
MOL005100	5,7-Dihydroxy-2-(3-hydroxy-4-methoxyphenyl)chroman-4-one	47.74	0.28	0.27	Zhishi
MOL005828	Nobiletin	61.67	1.05	0.52	Zhishi
MOL005849	Didymin	38.55	0.6	0.24	Zhishi
MOL000006	Luteolin	36.16	0.19	0.25	Zhishi
MOL007879	Tetramethoxyluteolin	43.68	0.96	0.37	Zhishi
MOL009053	4-[(2S,3R)-5-[(E)-3-Hydroxyprop-1-enyl]-7-methoxy-3-methylol-2,3-dihydrobenzofuran-2-yl]-2-methoxy-phenol	50.76	0.03	0.39	Zhishi
MOL000358	Beta-sitosterol	36.91	1.32	0.75	Danggui
MOL000449	Stigmasterol	43.83	1.44	0.76	Danggui
MOL010672	Icosa-8,11,14-trienoic acid methyl ester	44.81	1.49	0.23	Laifuzi
MOL000359	Sitosterol	36.91	1.32	0.75	Laifuzi
MOL003975	Icosa-11,14,17-trienoic acid methyl ester	44.81	1.52	0.23	Laifuzi
MOL001494	Mandenol	42	1.46	0.19	Chuanlianzi
MOL001495	Ethyl linolenate	46.1	1.54	0.2	Chuanlianzi
MOL002045	Stigmasterol	43.41	1.35	0.76	Chuanlianzi
MOL002047	Melianone	40.73	0.46	0.81	Chuanlianzi
MOL002048	Nimbolidin D	30.38	−0.28	0.53	Chuanlianzi
MOL002053	Nimbolin A	32.11	0.52	0.34	Chuanlianzi
MOL002056	(E)-3-[(2S,3R)-2-(4-Hydroxy-3-methoxy-phenyl)-7-methoxy-3-methylol-2,3-dihydrobenzofuran-5-yl]acrolein	54.74	0.22	0.4	Chuanlianzi
MOL002058	40957-99-1	57.2	0.49	0.62	Chuanlianzi
MOL000098	Quercetin	46.43	0.05	0.28	Chuanlianzi
MOL010813	Benzo [a] carbazole	35.22	1.87	0.22	Muxiang
MOL010828	Cynaropicrin	67.5	−0.1	0.38	Muxiang
MOL010839	Lappadilactone	38.56	−0.12	0.73	Muxiang
MOL000211	Mairin	55.38	0.73	0.78	Muxiang
MOL000359	Sitosterol	36.91	1.32	0.75	Muxiang
MOL000449	Stigmasterol	43.83	1.44	0.76	Muxiang

## Data Availability

The first author should be contacted to request for the data (ravernyflun@outlook.com or +8615202257239).
